# Long-term growth of temperate broadleaved forests no longer benefits soil C accumulation

**DOI:** 10.1038/srep42328

**Published:** 2017-02-08

**Authors:** Yu-he Ji, Ke Guo, Shi-bo Fang, Xiao-niu Xu, Zhi-gao Wang, Shu-dong Wang

**Affiliations:** 1 State Key Laboratory of Severe Weather (LASW), Chinese Academy of Meteorological Science, Beijing 100081, China; 2 State Key Laboratory of Vegetation and Environmental Change, Institute of Botany, Chinese Academy of Sciences, Beijing 100093, China; 3 School of Forestry & Landscape Architecture, Anhui Agricultural University, Hefei 230036, China; 4 Department of Life Science, Anqing Normal University, Anqing 246133, China; 5 State Key Laboratory of Remote Sensing Science, Institute of Remote Sensing and Digital Earth, Chinese Academy of Science, Beijing 100101, China

## Abstract

It is widely recognized that the long-term growth of forests benefits biomass carbon (C) sequestration, but it is not known whether the long-term growth of forests would also benefit soil C sequestration. We selected 79 representative soil profiles and investigated the influence of the forest stand age on the soil C dynamics of three soil layers (0–10, 10–20 and 20–30 cm) in temperate broadleaved forests in East China. The results suggest that the soil C density in temperature broadleaved forests significantly changes with the stand age, following a convex parabolic curve. At an early stand age, the soil C density usually increases, reaching its peak value at a pre-mature stand age (approximately 50 years old). At later stand ages, the soil C density usually decreases. Therefore, our results reveal a turning point in the soil C density at a pre-mature stand age. The long-term growth of temperate broadleaved forests after pre-mature stand age no longer benefits soil C accumulation, probably promotes topsoil C loss. In addition, we found that the soil C density in the upper soil layer usually changes with the forest stand development more significantly than that in deeper soil layers.

Soil carbon (C) in forests has attracted much attention in recent years because its stability contributes to the mitigation of climate change^1^,^2^. It was discovered that the soil C pool in temperate forests appears to be stable under disturbances, such as logging, wind storms, and invasive species^3^,^4^. It is widely recognized that the soil C pool in forests varies dynamically with the stand age^5^,^6^, but there are disagreements on how this occurs, leaving forest managers with uncertainty on how to best update forests for optimal C sequestration in the soil.

Rothstein *et al*. (2004) observed a weak decline in the surface soil C content with the stand age in Michigan jack pine forests^7^. On the contrary, Fonseca *et al*. (2011) discovered that the soil C increased by 1.1 Mg ha^−1^ yr^−1^ (1 Mg = 10^6^g) over the stand age range of 4~20 years in secondary tropical forests in Costa Rica^8^. Chen *et al*. (2013) discovered that the soil C pool in a Chinese fir plantation declined at young stand ages and then re-accumulated C at the stand ages of 16 ~ 21 years^9^. Shi & Cui (2010) argued, by summarizing 70 publications, that the highest soil C accumulation rate occurred at stand ages of 10–20 years old^10^. By summarizing more than 100 publications, Yang *et al*. (2011) argued that the soil C pool did not undergo significant changes during forest stand development in most studies^11^. Therefore, it remains uncertain how the soil C changes in the long-term growth process of forests.

In China, secondary forests have expanded due to reforestation over the past half century. Seven national-scale forest investigations have been performed since the 1950 s, but focused only on the timber volume and forest C biomass, without considering the soil C^12^. National-scale soil investigations have also been performed, but unfortunately, they focused on the soil C in different soil types rather than for vegetation types^13^,^14^. Some studies estimated the soil C pool in Chinese forests based on process-based BIOME models^15^, but could not resolve the complicated relationship between the soil C and forest stand age. Thus, it is necessary to elucidate how the soil C changes with the forest stand age to provide scientific evidence for forest management.

In this paper, we investigated 79 representative soil profiles in temperate broadleaved forests in eastern China. The objectives were to uncover the relationship between the soil C sequestration and the stand age of temperate broadleaved forests to improve forest management.

## Results

### Change trend of soil C density with stand age

**Figure 1:**
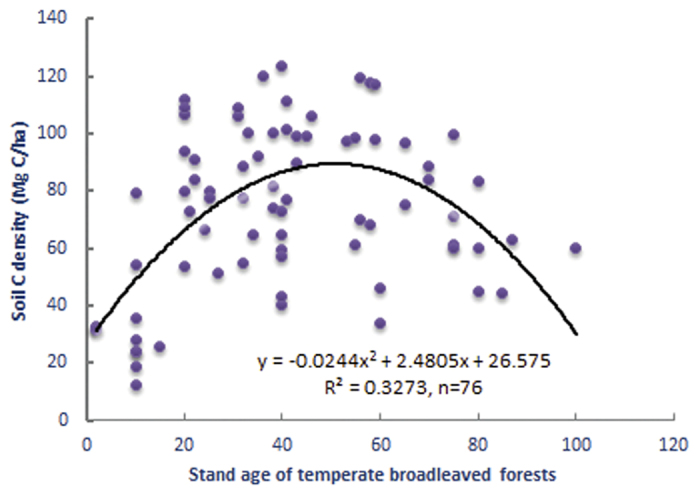
Soil C density change with actual stand age in soil layers (0–30 cm) in temperate broadleaved forests in Anhui Province, East China.

Regardless of the broadleaved tree species, we use the actual forest stand age as the independent variable to examine how the soil C density changes in three soil layers (0–10, 10–20, 20–30 cm) in temperate broadleaved forests. When the three soil layers were taken as a unit, the results show that the soil C density is significantly correlated with the forest stand age. The soil C density changes with the stand age following a convex parabolic curve (R^2^ = 0.3273), not a straight line. The soil C density increases at a young stand age, reaches its maximum carbon storage at an average age of approximately 50 years, and then gradually declines with the increasing stand age (Fig. 1). Therefore, the results indicate that there exists a turning point of soil C density in temperate broadleaved forests during stand age development. The soil acts as a C sink following forest establishment, but switches to a C source at approximately 50 years old, implying that the long-term growth of temperate broadleaved forests after 50 years no longer benefits soil C accumulation, but rather contributes to C loss from the soil.

**Figure 2:**
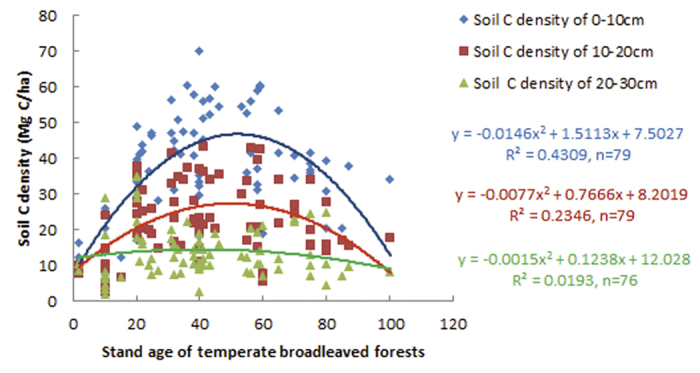
Soil C density change with actual stand age in three soil layers (0–10, 10–20, 20–30 cm) in temperate broadleaved forests in Anhui Province, East China.

When comparing the three soil layers, a significant change in the soil C density with the forest stand age following a parabolic curve was observed (R^2^ = 0.4309) in the upper soil layer (0–10 cm). In the soil layer of 10–20 cm, the soil C density also varied in a parabolic curve with the forest stand age (R^2^ = 0.2346), but the peak of the parabolic curve became lower. In the deeper soil layer of 20–30 cm, the peak of the parabolic curve disappeared (R^2^ = 0.0193), as the soil C density changed only slightly compared to in the upper soil layers (Fig. 2). As a result, the soil C in the upper layers is more sensitive to the forest stand age than that in the lower soil layers.

### Average change rate of soil C density with stand age class

**Figure 3:**
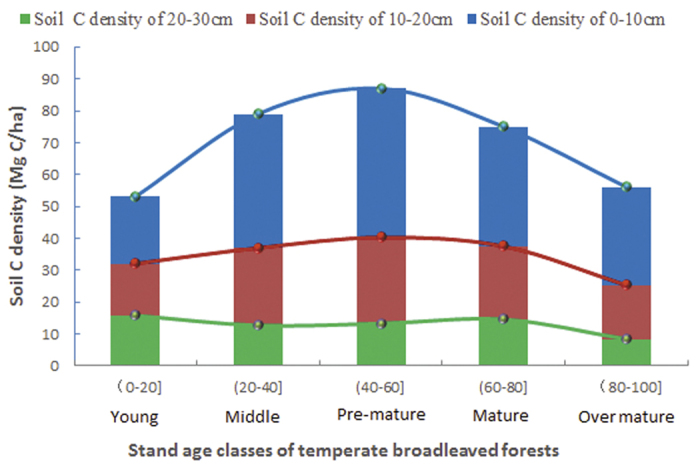
Soil C dynamics with stand age class in three soil layers (0–10, 10–20, 20–30 cm) in temperate broadleaved forests.

To quantify the soil C dynamics with the stand age class, we divided the entire growth sequence of temperate broadleaved forests into five stand age classes (young, middle, pre-mature, mature and over-mature) (Table 1). When the three soil layers (0–10, 10–20, 20–30 cm) were taken into account as a whole, the results suggest that the soil C density reaches its peak value (approximately 85.6 Mg C/ha) at the pre-mature stand age. On average, the soil C density increased at a rate of 0.813 Mg C/ha per year prior to the pre-mature stand age. Subsequently, it declined at a rate of 0.74 Mg C/ha per year after the pre-mature stand age and to 56.0 Mg C/ha at the over-mature stand age (average 91 years old). Therefore, the quantitative results indicate that the pre-mature stand age (average 52 years old) is a turning point in the soil C dynamics. The soil C accumulated at a rate of 0.813 Mg C/ha per year before the turning point and then exhibited a loss of 0.74 Mg C/ha per year after the turning point (Fig. 3).

Comparing the three soil layers, the upper soil layer (0–10 cm) showed the most significant change in soil C density, with an increase of 0.643 Mg C/ha per year from young to pre-mature and a decrease of 0.398 Mg C/ha per year from pre-mature to over-mature. The middle soil layer (10–20 cm) showed an increase of 0.268 Mg C/ha per year from young to pre-mature and a decrease of 0.253 Mg C/ha per year from pre-mature to over-mature. The deepest soil layer (20–30 cm) showed a slightly fluctuating soil C density without any significant change over the entire growth sequence.

## Discussion

**Figure 4:**
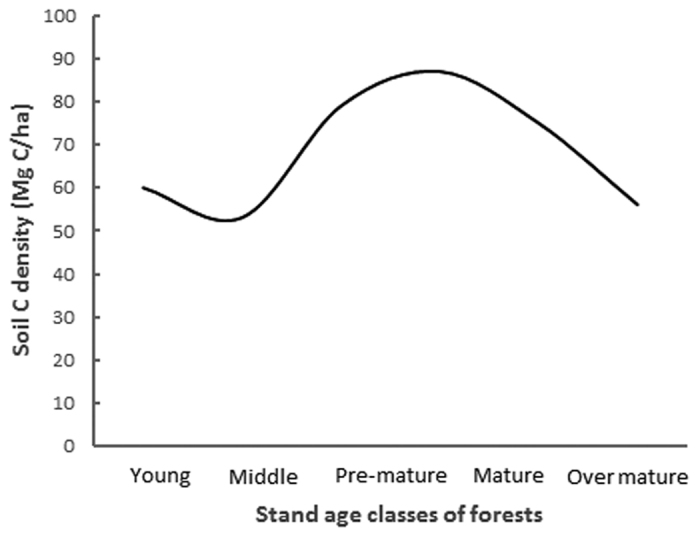
Multi-peak curve of topsoil C density change with forest stand age according to our study and other studies.

Our results demonstrate that the soil C density in temperate broadleaved forest changes with the stand age following a convex parabolic curve, and there exists a turning point with a single peak in the soil C accumulation at approximately 50 years old (Figs 1, 2 and 3). However, the results only show a general (significant single-peak curve) change trend of soil C density, but omits minor changes along the successional process since the data is not enough to identify the minor changes. The soil C density probably changes following a multi-peak curve with no more than one turning point during the entire forest stand age, as some studies reported that the soil C initially decreased or increased slowly for the first decade after afforestation and then began to accumulate quickly with the stand age. The multi-peak curve of soil C density usually exist under the precondition of afforestation. For example, Paul *et al*. (2002) synthesised available world-wide information on changes in soil C after afforestation, and argued that soil C in surface soil (<10 or <30 cm depth) initially declines during the first 5 years after establishing a plantation but recovers by the age of 30 years^16^. The initial decline of soil C in Paul *et al*. (2002) came from the average data in the 43 published or unpublished studies, so the decline is not significant since the data are highly variable, with soil C either increasing or decreasing in young (<10 year) forest stands. Li *et al*. (2011) observed the total mineral soil C initially appeared to decline at the early stand age, but recovered by the stand age of 35 years for coniferous plantation forest with Korean Pine (*Pinus koraiensis*). It is a pity that the natural change of soil C is not credible after 35-year-old stand because the soil C suffered from disturbance greatly from human, such as thinning treatment to the35- and 51-year-old stands^17^. Hiltbrunner *et al*. (2013) examined the effects of afforestation with Norway spruce (*Picea abies* L.) in a grazed subalpine pasture in Switzerland on soil organic carbon (SOC), and discovered that soil C stock decreased after tree establishment, reaching a minimum 40–45 years after afforestation, and increased thereafter^18^. Barcena *et al*. (2014) analyzed the changes in SOC stocks at the 0–30 cm soil layer following afforestation in Northern Europe by a meta-analysis, revealed that SOC loss generally for barren, cropland, heathland and grass-land at the initial phase following afforestation (0–30 years). The detectable gains in SOC stocks appear in later stages (>30 years), especially for afforestation of croplands^19^. Yu *et al*.^20^ investigated the soil C in four Chinese fir (*Cunninghamia lanceolata* Hook) plantations (Chinese fir was planted in clear-cut sites in natural broad-leaved forest) in Jiangxi Province, south China, discovered that soil C density at the depth of 0–20 cm declined before 16 years, but increased after 16 years, since soil C density declined from 35.98 Mg·ha^−1^ in the 7-year plantation to 30.12 Mg·ha^−1^ in the 16-year plantation, and then increased after 16 years old^20^. Therefore, we can speculate that soil C density probably changes following a multi-peak curve, and another turning point of the soil C density may exist in the early decades of afforestation, besides the large turning point at approximately 50 years old (Fig. 4).

In the later stage of forest development, many reports indicate that old forests are expected to maintain their biomass accumulation for a long time through the development of a multilayer canopy structure^21^, but this does not mean that the soil C can continue to increase as long as the biomass accumulates. Our results show that old-growth forest could not sustain the soil C increase due to the decreasing soil C density after the pre-mature stand age (average stand age 50 years old). However, there seems to be some controversy on this point. It is conventionally accepted that the soil C levels in old-growth forests are in a steady state^21^,^22^. However, Zhou *et al*. (2006) reported that soils in the top 20-cm soil layer in preserved old-growth forests (age > 400 years) in southern China accumulated C significantly at an unexpectedly high rate from 1979 to 2003^23^. Li & Liu (2014) argued that an old forest (38–56 y) was able to continuously accumulate C in the soil in China’s Loess Plateau, even when the biomass significantly decreased^24^.

These different opinions could be partly attributed to the different definitions of “old forest” with tree species and environment spatial variability, as there is currently no recognized definition. For example, a stand age of 38–56 years old in the study of Li & Liu (2014) was regarded as old forest^24^, equivalent to the pre-mature stands age (40–60 years old) in our study. It is likely that tree species and environment spatial variability are the leading causes of the different opinions, as different environments and tree species can affect the carbon accumulation-and-release processes^11^,^25^,^26^,^27^. The synergistic effects of many factors should be further explored to uncover the complex mechanism of soil C dynamics^28^,^29^,^30^,^31^.

## Conclusions

Our results show that soil C in temperature broadleaved forests significantly changes with stand age. Generally, it exhibits a change trend in the shape of a convex parabolic curve with stand age, regardless of the tree species. At the early stage of forest development, the soil C density usually increases, and it reaches its peak value at the pre-mature stand age (approximately 50 years old). At later stages of forest development, the soil C density usually decreases. This phenomenon provides strong evidence that there is a turning point of the soil C density in temperate broadleaved forests at the pre-mature stand age, when the soil switches from being a net C sink to a net C source. Therefore, we drew the conclusion that the long-term growth of temperate broadleaved forests after pre-mature stand age no longer benefits soil C accumulation. Our study also confirmed that the soil C in the upper layers is more sensitive to forest stand age than that of the lower soil layers, as the soil C density in the upper soil layers usually changes significantly with the forest stand development.

## Materials and Methods

### Study area

**Figure 5:**
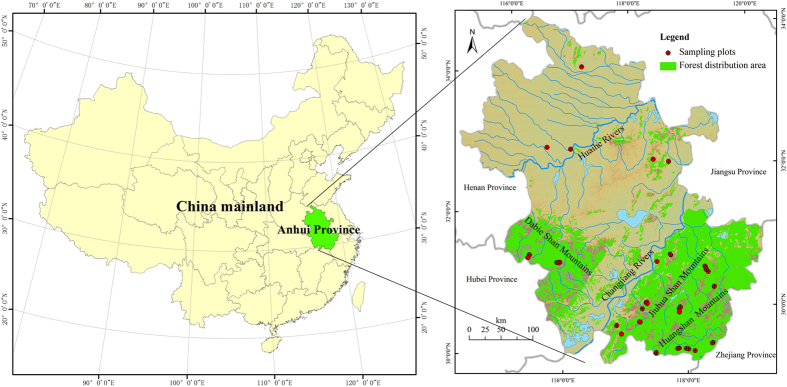
Location of study area and sampling plots of 79 representative soil profiles in temperate broadleaved forests in Anhui Province, East China (The map was generated using ArcGIS for Desktop 10.2, http://www.esri.com/software/arcgis).

The study area covers approximately 139,000 km^2^ in the Anhui Province (114°51′–119°36′E, 29°26′–34°37′N) of East China (Fig. 5). The Asian monsoon circulation creates a temperate continental monsoon climate with an annual average temperature of 14–17 °C and an annual precipitation of 800–1800 mm. The soil type is yellow brown soil^32^. Three mountain ranges (Dabie, Jiuhua and Huang Mountains) lie in the southwestern and southern regions (Fig. 5) and are covered with temperate and subtropical forests. The dominant forest types are temperate deciduous broadleaved forests, coniferous forests, and mixed forests.

In recent decades, large-scale deforestation has been curbed, and reforestation projects have been carried out, providing an opportunity to resume normal forest development^33^. Forest managers also allow people to update some forests to obtain timber for money. Therefore, the study area contains various forests with stand ages ranging from 0 to 100 years. However, it is unclear what updating schedule for the forest is the most favourable for soil C sequestration.

### Sampling sites (plots)

To avoid the effect of the spatial heterogeneity of sampling sites on SOC, we did our utmost to select coincident sampling sites (plots) in vegetation composition, soil type, and the same development process. All the sampling sites must have typical temperate broadleaved forest which is determined by climatic zones, though there are other forests, such as coniferous forests, conifer and broadleaf mixed forests. To avoid of human disturbance, all the sampling sites were selected in protecting natural forests to ensure a natural growth process. Young broadleaved forests being selected should have similar land use process because SOC in young forests suffer more effect from previous land use. We didn’t consider the young forests which land use type had been changed greatly by human. Thus, almost all forest vegetation in sampling sites belongs to secondary successional vegetation under the protection of human beings.

We selected the typical forests for every stand ages (young, middle, pre-mature, mature, over-mature) according to the natural succession of temperate broadleaved forest, so there is a slightly inconsistent in vegetation (species) composition for different stand age due to the natural succession. The vegetation composition for different stand ages is listed as follows (Table 2).

The soil in study area is classified as yellow brown soil zone according to “Map of Soil Regionalization of China”^34^. The sampling sites in our study ensured a typical yellow brown soil, and other soil types were avoided.

Generally, the sampling sites are coincident approximately in vegetation composition, soil type and development process, in spite of existing spatial heterogeneity more or less.

### Soil sampling

To examine the dynamics of the soil C with stand age, 79 soil profiles of sampling sites were investigated in representative temperate broadleaved forests in September of both 2011 and 2012 (Fig. 5). The actual stand age of each forest type was recorded by visiting farmers and forest management staff. The investigation focused on soil carbon density in the 0–30 cm soil layer, since soil carbon in the layer accounts for the majority of the soil profile 0–100 cm, and is sensitive to forest stand ages more than that in deeper soil layers. Vertical soil profiles were dug in the sampling plots, and soil samples were collected from three soil layers (0–10, 10–20, 20–30 cm) using a ring knife (volume of 100 cm^3^) for measuring the soil bulk density and a spade for measuring the soil C content. Soil samples from the surface soil layer (0–10 cm) included the forest floor (O horizons, i.e., the organic horizon), but surface litter was not included in the calculation of the soil C. In addition, the relevant environmental information for each sampling plot was recorded, such as the geographical location with latitude and longitude, forest type and stand age.

### Data analysis

The soil samples collected with the ring knife were used to measure the soil bulk density by the drying method in the laboratory. The soil samples collected with the spade were air-dried, ground using a mortar, and passed through a 2-mm sieve to remove all roots and stones. Finally, chemical analyses were performed to measure the soil C concentration by dry combustion using an elemental analyser (vario MACRO cube, Elementar, Germany).

For these soil profiles, we calculated the soil C density (*D*) of the three soil layers of 0–10, 10–20 and 20–30 cm using Equation (1),


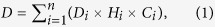


where *D* is the soil C density (10^5^ g C/ha.), *D*_*i*_ is the bulk density (g/cm^3^), *H*_*i*_ is the soil depth (cm), *C*_*i*_ is the soil C concentration (‰), and *i* represents the three soil layers.

## Additional Information

**How to cite this article**: Ji, Y.- *et al*. Long-term growth of temperate broadleaved forests no longer benefits soil C accumulation. *Sci. Rep.* **7**, 42328; doi: 10.1038/srep42328 (2017).

**Publisher's note:** Springer Nature remains neutral with regard to jurisdictional claims in published maps and institutional affiliations.

## Figures and Tables

**Table 1:** Soil C density at a depth of 0–30 cm in temperate broadleaved forests.

Period of stand age	Stand age classes	Average stand age	Number of soil profiles	Soil C density of 0–10 cm (Mg C/ha)	Soil C density of 10–20 cm (Mg C/ha)	Soil C density of 20–30 cm (Mg C/ha)
Young	(0–20]	12	18	21.0 ± 12.5	16.3 ± 10.6	15.8 ± 11.1
Middle	(20–40]	33	26	42.0 ± 11.5	24.3 ± 8.2	12.7 ± 4.4
Pre-mature	(40–60]	52	20	46.7 ± 13.0	27.0 ± 11.1	13.3 ± 3.7
Mature	(60–80]	74	11	37.5 ± 7.7	22.9 ± 7.0	14.6 ± 7.2
Over mature	(80–100]	91	3	30.8 ± 7.4	16.9 ± 0.9	8.4 ± 1.1

Note: stand ages refer to the standards of the “Forest Resource Statistics of China” (Zhang *et al*.^13^).

**Table 2:** The vegetation composition in sampling sites for different stand ages of temperate broadleaved forests in Anhui Province.

	Young forest	Middle age forest	Pre-mature forest	Mature and over mature forest
Tree layer	*Quercusglandulifera* var. *brevipetiolata, Pistaciachinensis,Broussonetiapapyrifera,Sorbushemsleyi*	*Quercusglandulifera* var. *brevipetiolata, Cyclobalanopsisglauca, Qercusacutissima, Castanopsiseyrei, Castanopsissclerophlla, Fraxinusinsularis*	*Quercusglandulifera* var. *brevipetiolata, Cyclobalanopsisglauca,Qercusacutissima, Castanopsiseyrei, Castanopsissclerophlla, Platycaryastrobilacea*	*Quercusglandulifera* var. *brevipetiolata, Cyclobalanopsisglauca, Qercusacutissima, Castanopsiseyrei*
Shrub layer	*Rhododendron simsii, Lespedeza viatorum, Linderareflexa, Zanthoxylumarmatum, RhizomaSmilacis*	*Rhododendron simsii, Lespedeza bicolor, Lorpetalumchinense, Linderaglauca, Glochidionpuberum,Camellia fraternal, Rhamnusglobosa*	*Rhododendron simsii, Lespedeza Formosa,Lorpetalumchinense,Camelliafraterna*	*Pleioblastusamarus, Camellia fraternal, Linderaglauca*
Herb layer	*Dryopterischinensis, Carextristachya,Oplismenusundulatifolius, Arthraxonhispidus, Commelinabengalensis, Carpesiumabrotanoides, Artemisia lavandulaefolia, Leonurus japonicas, Viola concordifolia*	*Dryopterischinensis, Woodwardia japonica, Carextristachya,Oplismenusundulatifolius, Rhizomaimperata, Aster ageratoides, Antenoronfiliforme, Viciaamoenafisch*	*Dryopterischinensis, Woodwardia japonica, Carextristachya, Oplismenusundulatifolius, Lindera aggregate, Phaenospermaglobosa*	*Dryopterischinensis, Woodwardia japonica, Carexbreviculmis, Oplismenusundulatifolius, Lophatherumgracile, Linderaaggregata*
